# Analysis of Treatment Patterns and Outcomes Among Patients with Third-Line or Later (3L+) Diffuse Large B-Cell Lymphoma (DLBCL) with Multiple Qualifying Index Dates Treated in the United States

**DOI:** 10.3390/jcm15197596

**Published:** 2026-09-30

**Authors:** Michael Wallington, Yong Chen, Fei Jie, Monica Patterson, Karen Repetny, Michelle Fanale, Simon Purcell, Jacob Ambrose, Christina M. Zettler, Andrew J. Belli, Laura L. Fernandes, Ching-Kun Wang

**Affiliations:** 1 Pfizer, Ltd., Walton Oaks, Tadworth KT20 7NS, UK; 2 Pfizer, Inc., Collegeville, PA 19426, USA; 3 Pfizer, Inc., Bothell, WA 98021, USA; 4 Verana Health (Formerly COTA, Inc.), New York, NY 10014, USA

**Keywords:** DLBCL, R/R, survival, line of therapy, outcomes

## Abstract

**Background/Objectives**: Diffuse large B-cell lymphoma (DLBCL) is an aggressive hematologic malignancy with a defined, first-line (1L) standard of care (SOC). Several novel agents have been approved for the treatment of relapsed/refractory (R/R) disease in recent years, leading to a heterogeneous treatment landscape in later lines of therapy (LOTs). Our study aimed to characterize real-world treatment patterns for patients who received third-line or later (3L+) therapy using a multiple index date qualification methodology to enable a comparison to clinical trials. **Methods**: This observational, retrospective study identified eligible patients in the COTA (now Verana Health) electronic health record-based, DLBCL dataset. Patients were assessed for eligibility at the initiation of each 3L+ LOT. The results were summarized overall and by qualification methodology, and the Kaplan–Meier method was used to evaluate real-world overall survival (rwOS). **Results**: Among 476 unique patients, 716 index-LOTs were identified, and 3L+ index therapies were variable. The Eastern Cooperative Oncology Group (ECOG) score was 0–2 for 57% of qualifying-LOTs and missing/unknown (M/U) for 43%. The median rwOS among all-qualifying, first-qualifying, and last-qualifying index-LOTs was 8.0 (95% CI: 6.9, 10.6), 11.4 (95% CI: 9.7, 13.6), and 6.3 months (95% CI: 5.3, 8.0), respectively. Patients with M/U ECOG did not experience worse outcomes compared to those with ECOG 0–2. **Conclusions**: The outcomes in our study are similar to those from comparator arms of contemporary clinical trials, demonstrating a continued unmet need for effective, SOC therapy for patients with 3L+ DLBCL. Additionally, the variable outcomes based on qualifying index-LOTs underscores the criticality of appropriate patient identification and comparison for clinical trials.

## 1. Introduction

Diffuse large B-cell lymphoma (DLBCL) is an aggressive form of non-Hodgkin lymphoma (NHL), marked by rapidly growing tumors in lymph nodes, spleen, liver, bone marrow, and/or other organs [1]. For newly diagnosed patients, the first-line (1L) standard of care (SOC) for standard risk disease is rituximab with cyclophosphamide, doxorubicin, vincristine, and prednisone (R-CHOP), or the recently approved polatuzumab vedotin in combination with rituximab, cyclophosphamide, doxorubicin, and prednisone (Pola-R-CHP) [2,3]. While these regimens are highly effective, approximately half of patients experience relapsed or refractory (R/R) disease [4].

Chimeric antigen receptor (CAR) T-cell therapy or high-dose chemotherapy with hematopoietic stem cell transplant (HSCT) are recommended second-line (2L) treatments. Due to persistent access issues, the 2L treatment landscape is less standardized compared to 1L [5]. The third-line and later (3L+) treatment landscape, however, is even more heterogenous with numerous treatment options available, including CAR-T cell therapy, antibody-drug conjugates (i.e., pola or loncastuximab), monoclonal antibodies (i.e., tafasitamab), or bispecifics (i.e., glofitamab or epcoritimab), among other agents. Treatment selection varies and is based on patient fitness or preference, provider and/or institution preference, patient access, and other factors [6].

The heterogenous nature of this treatment landscape has an impact on R/R DLBCL clinical trials as a single SOC is not well-defined, therefore presenting challenges for comparator selection. As numerous novel agents are under investigation in the 3L+ setting, real-world data are helpful to contextualize clinical trial results for comparable patients being treated in routine clinical practice. Real-world studies mimicking trial qualification and enrollment with stringent inclusion and exclusion criteria and comparable qualification methodologies have been constructed to provide an appropriate comparison to the trial results; however, these methods are often imperfect, necessitating an additional investigation into innovative qualification methodologies and comparisons. Importantly, in clinical trials, a patient qualifies only once at trial enrollment; however, the patient may be trial-eligible multiple times in the treatment journey (i.e., in lines of therapy (LOTs) prior to or following their enrollment date) depending on when the trial assessment occurs. The singular qualification date does not apply when analyzing retrospective real-world data. Rather, it is possible to assess patients across multiple qualification dates (i.e., index dates). As such, these studies ultimately require a deliberate decision to determine which qualification date will be used for the analysis and, ultimately, comparison to clinical trial populations.

The ECHELON-3 trial (ClinicalTrials.gov identifier: NCT04404283) assessed brentuximab vedotin (BV), lenalidomide, and rituximab (R2) compared to R2 alone for patients with R/R DLBCL who were not eligible for CAR-T cell therapy or HSCT [7]. Patients who received R2 were heavily pretreated with a median of three prior LOTs received. The median overall survival (OS) among patients in the BV and control arms were 13.8 (95% CI: 10.3, 18.8) and 8.5 months (95% CI: 5.4, 11.7), respectively, demonstrating a significant improvement in OS for this cohort [7]. Due to the lack of clear SOC in the 3L+ setting, additional research is needed to understand real-world care patterns and outcomes among this patient population.

In this study, we aim to evaluate real-world 3L+ treatment patterns among patients similar to those enrolled in the ECHELON-3 trial, namely, patients who do not receive CAR-T cell therapy or HSCT at qualification or enrollment. We assessed real-world outcomes among this cohort using a multiple index date qualification methodology to allow for comparisons to trial populations for ECHELON-3 and relevant clinical trials studying a similar R/R DLBCL population.

## 2. Materials and Methods

This study was an observational, retrospective study of records from COTA’s (now Verana Health) electronic health record (EHR)-based Vantage DLBCL database. Patients were assessed for eligibility at the initiation of each 3L+ therapy. Qualifying index-LOTs (multiple per patient aged ≥ 18 years) were identified by initiation of 3L+ therapy between 18 October 2017 (the approval date of the first CAR-T cell therapy in this indication in the US) and 31 December 2023 (to allow for approximately 8 months of minimum potential follow-up time from data lock date of 30 August 2024). Index LOTs were excluded if any of the following criteria were met: documentation of an Eastern Cooperative Oncology Group (ECOG) score of ≥3 at baseline, history of solid tumor malignancy within two years prior to index date, central nervous system (CNS) involvement prior to first-qualifying index date, exclusionary treatment history (brentuximab vedotin in any prior LOT or at index, CAR-T cell therapy or HSCT at index, or any clinical study therapy at index), or insufficiently precise key dates (as applicable: date of diagnosis, date of death, date of last contact, date of diagnosis of second primary malignancy, or index date). ECHELON-3 trial criteria were mapped to COTA’s real-world database (Table S1).

The index date(s) were defined as initiation of eligible 3L and/or subsequent LOTs. All-qualifying index-LOTs were included as index dates, and analyses were conducted among all-qualifying index-dates (multiple per patient), first-qualifying index date (one per patient), and last-qualifying index date (one per patient). Within each qualification approach, analyses were conducted by LOT and by ECOG score at baseline (ECOG 0–2 vs. missing/unknown (M/U)). The baseline period for time-varying covariates (such as ECOG score and labs) began at the earlier of (a) 30 days prior to index or (b) the end of prior LOT not exceeding 90 days prior to index. The baseline period continued until 7 days after index to allow for delay in documentation in the health record. If multiple results were present, the result closest to the index date was prioritized. If multiple results were present on the closest date, the worst result was prioritized. For molecular markers, any result from prior to diagnosis until +7 days from the index date was considered, and positive results were prioritized over negative.

Characteristics and treatment patterns were summarized by qualification methodology and among subgroups using patient counts and proportions, mean, standard deviation, median, and interquartile range (IQR), as appropriate. The Kaplan–Meier (KM) method was used to evaluate real-world OS (rwOS; defined as time from index date to date of death; patients without a date of death were censored at the last clinically relevant date) and time to next treatment or death (rwTTNTD; defined as time from index date to the initiation of the next LOT or death; patients without an event were censored at the later of last contact with a healthcare provider or diagnosis of second primary malignancy due to the inability to perfectly ascribe treatment, response, and progression information to one cancer or the other). Reverse KM method was used to evaluate follow-up. Robust variance estimation was used to account for the within subject correlation for endpoint estimates (i.e., overall rwOS and rwTTNTD) caused by having multiple observations per patient. All analyses were performed using R statistical software (version 4.5.1; R Core Team, Vienna, Austria).

## 3. Results

### 3.1. Characteristics and Treatment Patterns

A total of 716 qualifying index-LOTs were identified among 476 unique patients (Table 1); 399, 173, 73, 37, and 20 qualifying LOTs were included in the analysis in 3L, 4L, 5L, 6L, and 7L, respectively (Figure 1). Additional records qualified in 8L+; however, fewer than 10 records qualified per LOT, so those results are masked for privacy reasons. Among all-qualifying index-LOTs, the median age at diagnosis and at index date was 64 and 66.5 years, respectively (Table 1).

The majority of unique patients were male (58.8%), White (69.3%), non-Hispanic (67.0%), and treated in the community setting (65.5%). The proportion of patients treated in an academic setting was higher among later index LOTs (3L: 31.8% academic setting; 5L: 46.6%; 7L: 60.0%) (Table 2).

Among all-qualifying index-LOTs, patients were most frequently diagnosed in 2014 or later (86.2%), although patients were diagnosed as early as 1994 (Table 1). The calculated International Prognostic Index (IPI) score at diagnosis was highly missing (68.7%) due to the missingness of the data components, namely, the ECOG score at diagnosis (57%), LDH at diagnosis (50%), extranodal disease at diagnosis (31%), and stage at diagnosis (12%) (Table 1).

More than 70% of the 3L-qualified study population received SOC R-CHOP or similar regimens in 1L (Table 3). Following 1L, regimens became less standardized. ICE-related (17.8%) and bendamustine-containing regimens (10.5%) were most common in 2L. Among the 3L-qualified population, CAR-T cell therapy and HSCT were exclusionary at the index, so no patients received either cellular therapy in 3L. Pola-bendamustine + rituximab (Pola-BR)-related regimens (12.3%) and gemcitabine + oxaliplatin (GemOx)-related regimens (10.5%) were most common in 3L. CAR-T cell therapy was most common in 4L (28.2%) and 5L (23.2%). Similar trends were observed in the 4L+-qualified populations (Table 3). The most common treatment classes are summarized in Table 3, and all less-frequently administered classes within a given LOT are combined into “Other”, which could include investigational therapy or CD20-directed bispecific antibodies.

### 3.2. Outcomes

The median rwTTNTD among all-qualifying, first-qualifying, and last-qualifying index-LOTs were 3.3 (95% CI: 3.0, 3.7), 3.5 (95% CI 3.2, 4.0), and 3.6 months (95% CI: 3.3, 4.3), respectively (Table 4). Over a median follow-up time calculated using the Reverse KM method of 26.7 months (95% CI: 22.7, 33.7) among all-qualifying index-LOTs, there were 480 death events (Table 1 and Figure 2). The median rwOS among all-qualifying index-LOTs was 8.0 months (95% CI: 6.9, 10.6) (Table 4 and Figure 2). The median rwOS from the first-qualifying LOT and last-qualifying LOT were 11.4 (95% CI: 9.7, 13.6) and 6.3 (95% CI: 5.3, 8.0) months, respectively.

### 3.3. Subgroup Analysis

The qualifying index-LOTs with ECOG 0–2 (N = 408) and M/U (N = 308) at baseline had similar demographic characteristics, including age at diagnosis and index, sex and race distribution, and treatment setting (Table 5). Among patients with a missing ECOG score, there was generally a greater missingness in other data elements, especially within the molecular marker results. The distribution of results did not differ meaningfully by ECOG 0–2 versus M/U with the exception of years of diagnosis and index LOT initiation (index initiation 2021–2023: ECOG 0–2: 35.0%; M/U: 59.1%). Nearly one-third (30.5%) of patients with ECOG M/U at baseline had a known ECOG score of 0–2 at diagnosis, and an additional 4 patients (1.3%) had a known ECOG score of ≥3 at diagnosis. Almost half of the patients with a known ECOG score of 0–2 at baseline had an M/U ECOG score at diagnosis.

Over a median follow-up time (Reverse KM) of 36.1 (95% CI: 28.0, 41.5) and 17 months (95% CI: 15.0, 24.2), the median rwTTNTD for ECOG 0–2 and ECOG M/U was 3.3 (95% CI: 2.8, 3.7) and 3.4 months (95% CI: 3.0, 3.8), respectively (Table 5). The median rwOS for ECOG 0–2 and ECOG M/U was 6.9 (95% CI: 5.8, 9.1) and 10.6 months (95% CI: 7.9, 13.8), respectively, and the 30-month rwOS rate was 17% (95% CI: 14%, 22%) among the ECOG 0–2 group and 24% (95% CI: 18%, 32%) for the ECOG M/U group (Table 4 and Figure 3).

## 4. Discussion

In this study, a total of 716 qualifying index-LOTs were identified among 476 unique patients. The majority of qualifying index-LOTs occurred in 3L (n = 399; 55.7%), followed by 4L (n = 173; 24.2%), 5L (n = 73; 10.2%), 6L (n = 37; 5.2%), and 7L (n = 20; 2.8%) (Figure 1). In using the multi-index qualification methodology, we observed a 50% increase in qualifying index-LOTs relative to unique, qualified patients. The demographic and clinical characteristics of the overall population were similar to those in previously published studies of patients with DLBCL (Table 1). The median age at diagnosis for the study population was slightly younger than the general DLBCL population (64 years in this analysis; 67 years based on SEER data) [8]. This is likely due to the selection of younger and/or fitter patients into our study population due to the requirement to receive 3L+ therapy. The study population was also majority male (58.8% of unique patients), consistent with the available epidemiological data for DLBCL [9]. Two-thirds of the study population had Stage III or IV disease documented at diagnosis (although nearly 12% of the qualifying index-LOTs were missing staging information) (Table 1), consistent with the staging data from prior publications [6]. The year of DLBCL diagnosis spanned from 1994 to 2023. Patients diagnosed in more recent years that rapidly progressed and received 3L+ therapy may represent a cohort of patients with more aggressive disease characteristics, which may affect our results.

In this study, the treatment patterns were consistent with the available data, demonstrating that R-CHOP is the clear standard of care in the 1L setting and that treatment is increasingly more heterogeneous in later lines of therapy [10,11,12]. Notably, receipt of CAR-T cell therapy and/or HSCT was exclusionary atindex for this study population. As such, the proportion of patients that received these cellular therapies in a given index LOT is not reflective of broader real-world treatment patterns. For example, among the 3L-qualified population, 28.2% and 23.2% received CAR-T cell therapy in 4L and 5L, respectively. Among the 4L-qualified population, CAR-T cell therapy receipt was exclusionary at index (i.e., 4L), and the proportion of patients who received CAR-T cell therapy in 3L and 5L was 12.7% and 30.0%, respectively (Table 3). As such, these results should be interpreted with caution when assessing the real-world uptake of these cellular therapies by LOT.

Our study found that, despite the available treatment options, the median rwOS remains poor among patients with DLBCL receiving non-CAR-T cell therapy in 3L+. Sineshaw et al. (2024) found a median rwOS of 11.0 months (95% CI: 7.4, 20.8) from 3L initiation and 8.9 months (95% CI: 5.5, 12.0) from 4L initiation among R/R DLBCL patients [11]. Similarly, Crombie et al. (2024) identified a population of anti-CD20 monoclonal antibody-exposed R/R DLBCL patients, and those who received pola-based and tafasitamab-based regimens in 3L+ experienced a median rwOS of 7.4 months and 6.3 months, respectively [13]. These results are similar to those found in our study; however, all three studies used COTA’s DLBCL dataset, likely influencing the similarity of results.

Our findings support prior studies which found that real-world DLBCL treatment patterns are complex with no clear SOC in later-line settings [11,12]. Due to the varied nature of 3L+ treatment patterns for DLBCL, “usual” care encompasses many systemic regimens, and treatment selection may depend upon prior exposures, physician and/or institution preferences, and patient fitness and/or preference, among other factors. In the context of this heterogeneous treatment paradigm, it is clear that there is no single SOC for 3L+ DLBCL to be used as a comparator in ongoing clinical trials in this population. Approvals of novel agents during the study period (e.g., polatuzumab vedotin in 2019 (accelerated approval) or loncastuximab in 2021) further increased the heterogeneity of available treatment options [14,15]. Additional research is needed to stratify these results by the year of index LOT initiation to account for the changes in treatment paradigm, especially as it relates to outcome analyses.

The decision to use an all-qualifying index-LOTs approach stemmed from an important study design consideration for appropriate trial emulation: while a patient can enroll in a specific clinical trial at a single point in time, the patient, in actuality, may be eligible for the trial over multiple LOTs. In a retrospective study, analysts must make a deliberate choice of index date(s) for qualification. As such, we chose to allow multiple qualifying index-LOTs to assess the potential differences in outcomes resulting from the selection of more advantageous (i.e., earlier LOTs) or detrimental (i.e., last LOT) index dates. This enables a comparison to clinical trial results, such as ECHELON-3 (the median OS of the comparator arm: 8.5 months), in which the majority of patients in the control arm were heavily pre-treated with three or more LOTs prior to enrollment [7]. The approach to include all-qualifying index-LOTs had been previously examined and was found to be an appropriate strategy to define time zero [16,17,18].

In this analysis, the median rwOS for all-qualifying LOTs, first-qualifying LOT, and last-qualifying LOT was 8.0 (95% CI: 6.9, 10.6), 11.4 (95% CI: 9.7, 13.6), and 6.3 months (95% CI: 5.3, 8.0), respectively (Table 4). Considering only the first-qualifying index date may select for a less pre-treated and potentially younger population, which may have a positive impact on the outcomes for that cohort, while considering only the last-qualifying index date may select for a more heavily pre-treated and/or older population, potentially resulting in poorer outcomes for this cohort. Based on prior work related to time zero selection in observational datasets, allowing qualification multiple times for a single patient can be more statistically efficient and minimize bias in the cohort [16,17,18]. In the context of this heterogeneous treatment paradigm, the median rwOS between 6.3–11.4 months depending on the index date selection demonstrates that patients receiving the usual care in the US have a clear unmet need.

Additionally, the rwTTNTD in our study (all-qualifying index-LOTs: 3.3 months) was broadly similar to PFS in ECHELON-3 (the median PFS of the comparator arm: 2.6 months) [7] (Table 4). Due to limitations related to real-world progression capture and documentation, rwTTNTD is frequently used as a proxy for PFS under the assumption that physicians would not initiate new therapy in the real-world setting in the absence of disease progression [19]. Finally, the ECOG score at index was known for the majority (57%) of qualifying index-LOTs and was M/U for 43% of index-LOTs (Table 1). The median rwOS among all index-LOTs with ECOG 0–2 and M/U was 6.9 (95% CI: 5.8, 9.1) and 10.6 months (95% CI: 7.9, 13.8), respectively, although the median follow-up time and distribution of the year of index LOT initiation differed between groups (Table 4). Additional research is needed to assess if patients with the M/U ECOG score may have a comparable functional status to those with ECOG 0–2 and could be included in future clinical trial comparisons.

The strengths of this study are related to the contemporaneous nature of COTA’s dataset and the inclusion of patients treated in both academic and community oncology centers in the Northeast, Mid-Atlantic, Southern, and Western regions of the US. The limitations of this study are primarily those related to the use of real-world data. The accuracy and capture of data is limited by the documentation of those data in the EHR of the COTA-partnered center. Missingness may result from the lack of explicit documentation (i.e., tests performed but not recorded explicitly) or treatment received outside of the partnered center and records not transferred to the partnered center; however, any records provided to a partnered center and available within the EHR are available for abstraction.

Additionally, the study methodology (qualifying one patient multiple times at different LOTs) presents limitations. Due to the inclusion of individual records multiple times in the final all-qualifying LOTs cohort, intra-patient correlation can impact results. Robust variance estimation was used to account for this within-subject correlation. To additionally address this limitation, the results of the sensitivity analysis (among first- and last-qualifying LOT) are provided to contextualize the multi-qualification results. It was the authors’ intention to assess the inclusion of patients multiple times in the study population based on the various points in time at which the patient may qualify for the study. Because this analysis was descriptive in nature, the above approach was deemed appropriate, acknowledging the interpretation limitations. Some subgroups (e.g., 7L qualification cohort) are relatively small, and the results should be interpreted with caution. Finally, LOT is not routinely documented in real-world data. COTA uses a proprietary, disease-specific algorithm to assign LOT to abstracted data. While this algorithm has been reviewed by multiple internal and external experts, the perfect assignment of LOT is not guaranteed.

This study demonstrates the feasibility of using real-world data to contextualize the results reported from a clinical trial and highlights the absence of a singular later-line standard of care for patients receiving 3L+ therapy for DLBCL. There is a critical need for novel treatment options for patients with R/R DLBCL receiving 3L+ therapy who may be ineligible for HSCT and/or CAR-T cell therapy.

## Figures and Tables

**Figure 1:**
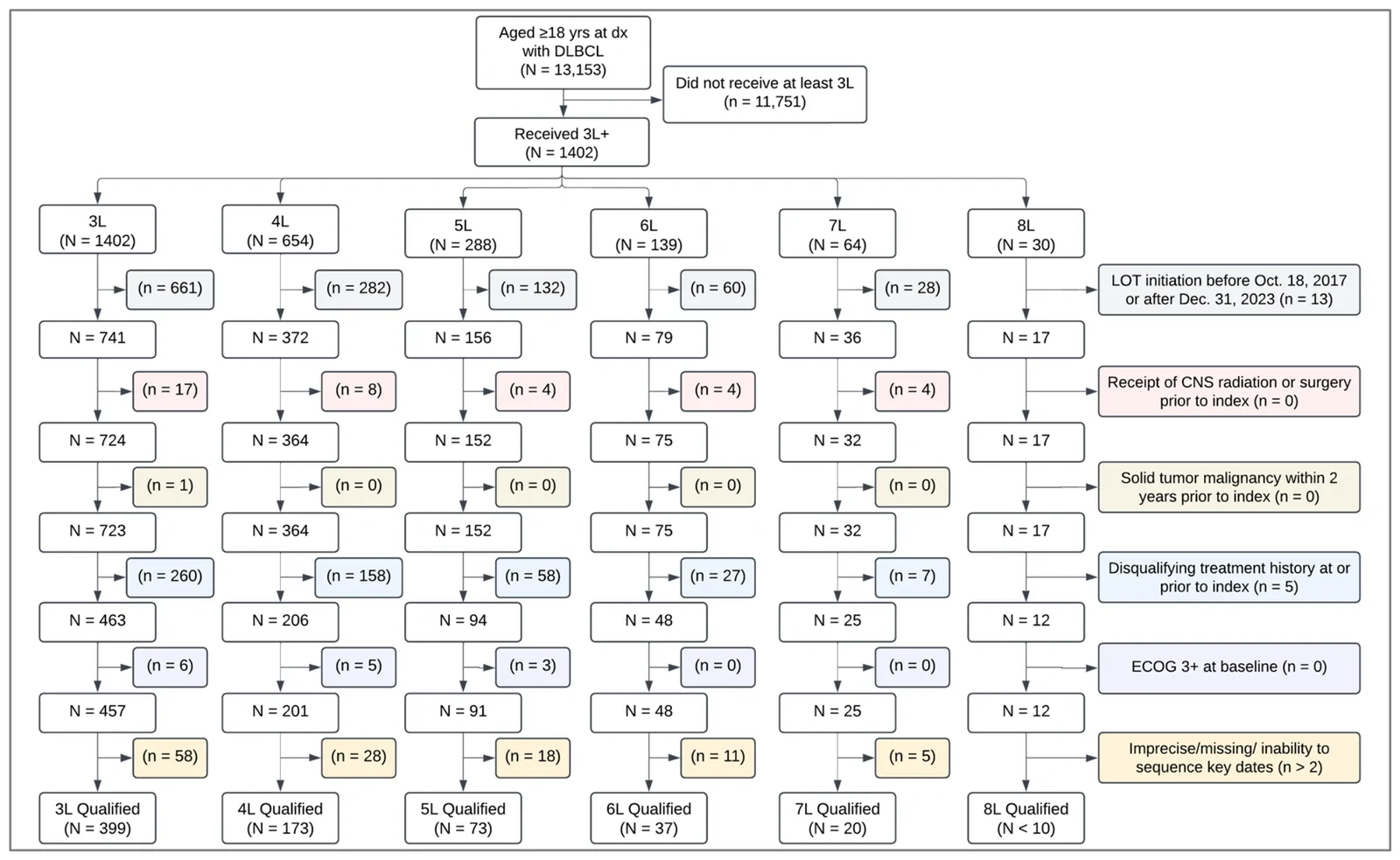
Attrition diagram.

**Figure 2:**
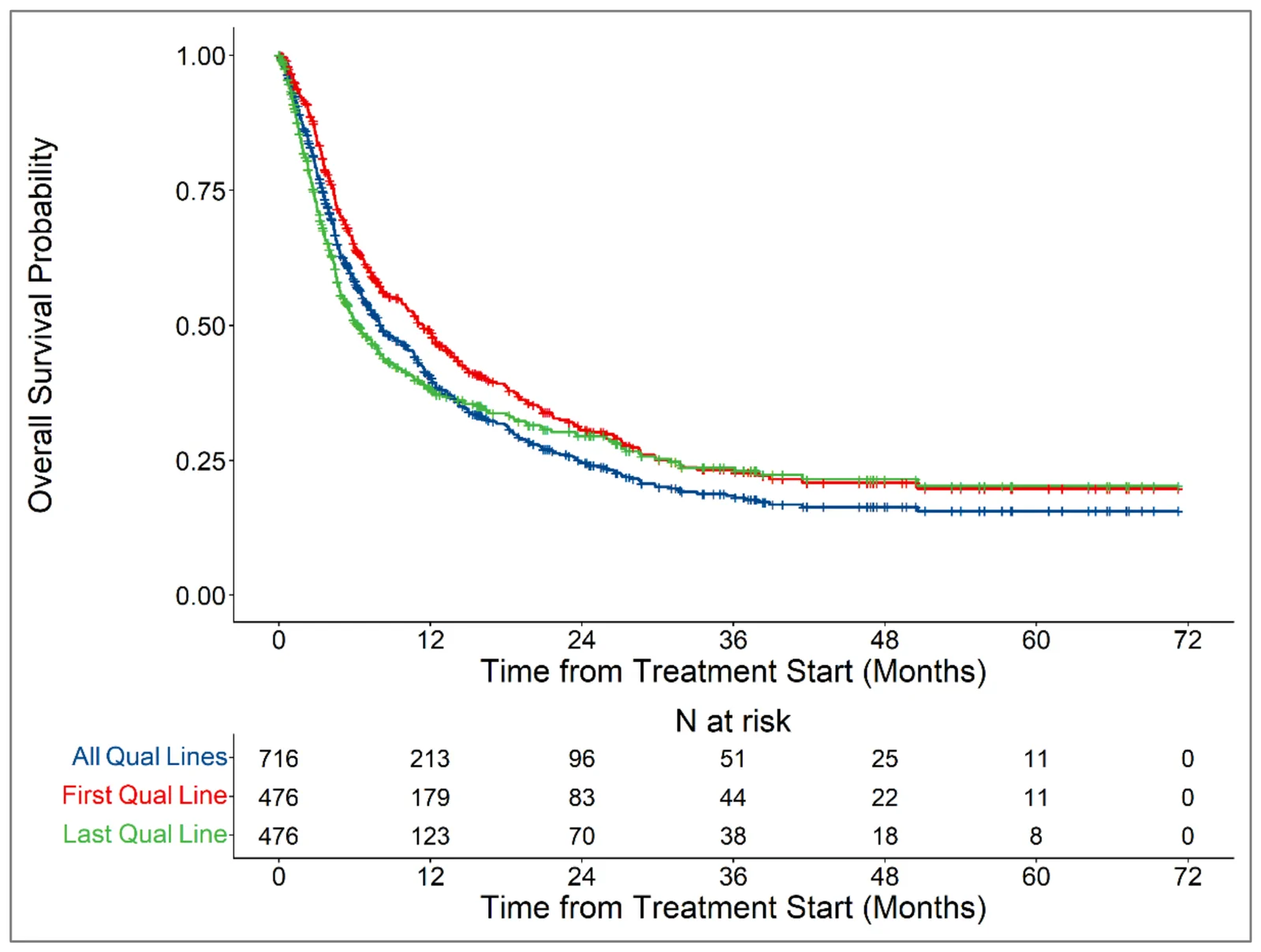
KM curve—rwOS among all-, first-, and last-qualifying LOTs.

**Figure 3:**
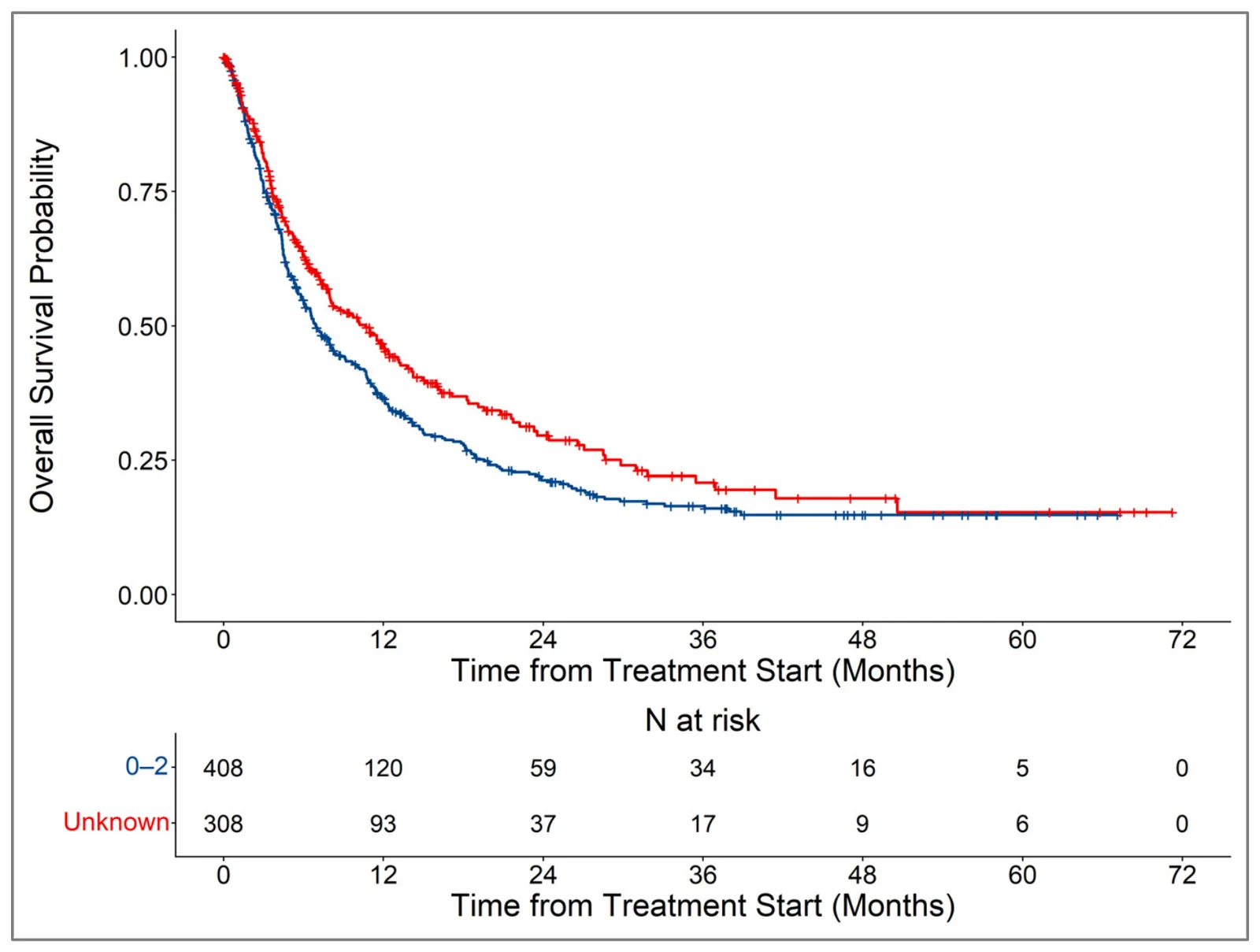
KM curve—rwOS among ECOG 0–2 vs. M/U for all-qualifying LOTs.

**Table 1:** Characteristics among all-qualifying, first-qualifying, and last-qualifying index-LOT.

	Overall (N = 716)	First-Qualifying LOT (N = 476)	Last-Qualifying LOT (N = 476)
Age at Diagnosis, median [IQR]	64.0 [54.0, 72.0]	64.0 [55.0, 73.0]	64.0 [55.0, 73.0]
Age at Index, median [IQR]	66.5 [57.0, 76.0]	67.0 [57.0, 76.0]	68.0 [57.0, 76.0]
Sex, n (%)			
Female	276 (38.5)	196 (41.2)	196 (41.2)
Male	440 (61.5)	280 (58.8)	280 (58.8)
Race, n (%)			
Asian	26 (3.6)	15 (3.2)	15 (3.2)
Black or African American	37 (5.2)	24 (5.0)	24 (5.0)
White	498 (69.6)	330 (69.3)	330 (69.3)
Other	135 (18.9)	89 (18.7)	89 (18.7)
Unknown	20 (2.8)	18 (3.8)	18 (3.8)
Ethnicity, n (%)			
Hispanic	207 (28.9)	140 (29.4)	140 (29.4)
Non-Hispanic	486 (67.9)	319 (67.0)	319 (67.0)
Unknown	23 (3.2)	17 (3.6)	17 (3.6)
Practice Setting, n (%)			
Academic	261 (36.5)	164 (34.5)	164 (34.5)
Community	455 (63.5)	312 (65.5)	312 (65.5)
Year of diagnosis, n (%)			
1994–2003	13 (1.8)	9 (1.9)	9 (1.9)
2004–2008	22 (3.1)	17 (3.6)	17 (3.6)
2009–2013	64 (8.9)	40 (8.4)	40 (8.4)
2014–2018	308 (43.0)	200 (42.0)	200 (42.0)
2019	111 (15.5)	75 (15.8)	75 (15.8)
2020	66 (9.2)	46 (9.7)	46 (9.7)
2021	71 (9.9)	52 (10.9)	52 (10.9)
2022	59 (8.2)	35 (7.4)	35 (7.4)
2023	2 (0.3)	2 (0.4)	2 (0.4)
Year of Index, n (%)			
2017	20 (2.8)	19 (4.0)	14 (2.9)
2018	110 (15.4)	78 (16.4)	67 (14.1)
2019	126 (17.6)	84 (17.6)	84 (17.6)
2020	135 (18.9)	90 (18.9)	85 (17.9)
2021	125 (17.5)	85 (17.9)	82 (17.2)
2022	103 (14.4)	61 (12.8)	70 (14.7)
2023	97 (13.5)	59 (12.4)	74 (15.5)
Follow-up time from diagnosis in months, median (IQR)	29.3 [17.0, 66.7]	30.4 [15.4, 67.6]	30.4 [15.4, 67.6]
Time from diagnosis to index in months, median (IQR)	19.4 [10.6, 43.4]	17.3 [9.9, 41.0]	20.2 [11.1, 46.6]
Follow-up time from index in months (Reverse KM), median (95% CI)	26.7 (22.7, 33.7)	27.5 (24.4, 33.7)	23.7 (19.7, 30.1)
ECOG at Index, n (%)			
0–2	408 (57.0)	263 (55.3)	271 (56.9)
≥3	0 (0.0)	0 (0.0)	0 (0.0)
Unknown	308 (43.0)	213 (44.7)	205 (43.1)
DLBCL Subtype, n (%)			
Activated B-cell–like	278 (38.8)	181 (38.0)	181 (38.0)
Activated B-cell–like and Germinal center B-cell–like	34 (4.7)	20 (4.2)	23 (4.8)
Germinal center B-cell–like	252 (35.2)	176 (37.0)	179 (37.6)
Unknown	152 (21.2)	99 (20.8)	93 (19.5)
Stage at Diagnosis by Any Staging System, n (%)			
1	26 (3.6)	21 (4.4)	21 (4.4)
2	121 (16.9)	79 (16.6)	79 (16.6)
3	194 (27.1)	120 (25.2)	120 (25.2)
4	291 (40.6)	196 (41.2)	196 (41.2)
Unknown	84 (11.7)	60 (12.6)	60 (12.6)
Calculated IPI at Diagnosis, n (%)			
Low Risk (IPI Score 0–1)	77 (10.8)	50 (10.5)	50 (10.5)
Low–Intermediate Risk (IPI Score 2)	90 (12.6)	62 (13.0)	62 (13.0)
High–Intermediate Risk (IPI Score 3)	41 (5.7)	28 (5.9)	28 (5.9)
High Risk (IPI Score 4–5)	16 (2.2)	13 (2.7)	13 (2.7)
Unknown	492 (68.7)	323 (67.9)	323 (67.9)
ECOG at Diagnosis, n (%)			
0–2	303 (42.3)	204 (42.9)	204 (42.9)
≥3	7 (1.0)	7 (1.5)	7 (1.5)
Unknown	406 (56.7)	265 (55.7)	265 (55.7)
Extranodal disease at Diagnosis, n (%)			
Present	487 (68.0)	314 (66.0)	314 (66.0)
Absent	5 (0.7)	4 (0.8)	4 (0.8)
Unknown	224 (31.3)	158 (33.2)	158 (33.2)
LDH at Diagnosis, n (%)			
<ULN	91 (12.7)	62 (13.0)	62 (13.0)
1–2x ULN	178 (24.9)	117 (24.6)	117 (24.6)
>2x ULN	90 (12.6)	60 (12.6)	60 (12.6)
Unknown	357 (49.9)	237 (49.8)	237 (49.8)
BCL2, n (%)			
Negative	73 (10.2)	48 (10.1)	45 (9.5)
Positive	576 (80.4)	380 (79.8)	390 (81.9)
Unknown	67 (9.4)	48 (10.1)	41 (8.6)
BCL6, n (%)			
Negative	102 (14.2)	68 (14.3)	63 (13.2)
Positive	552 (77.1)	367 (77.1)	375 (78.8)
Unknown	62 (8.7)	41 (8.6)	38 (8.0)
C-MYC, n (%)			
Negative	204 (28.5)	145 (30.5)	142 (29.8)
Positive	395 (55.2)	246 (51.7)	254 (53.4)
Unknown	117 (16.3)	85 (17.9)	80 (16.8)

**Table 2:** Characteristics among all-qualifying index-LOTs by LOT.

	3L (N = 399)	4L (N = 173)	5L (N = 73)	6L (N = 37)	7L (N = 20)
Age at Diagnosis, median [IQR]	65.0 [56.0, 73.0]	64.0 [54.0, 71.0]	60.0 [51.0, 71.0]	60.0 [51.0, 67.0]	56.5 [55.5, 64.0]
Age at Index, median [IQR]	68.0 [58.0, 76.0]	67.0 [57.0, 75.0]	64.0 [55.0, 74.0]	66.0 [54.0, 70.0]	63.0 [57.0, 66.2]
Practice Setting, n (%)					
Academic	127 (31.8)	66 (38.2)	34 (46.6)	16 (43.2)	12 (60.0)
Community	272 (68.2)	107 (61.8)	39 (53.4)	21 (56.8)	8 (40.0)
Year of Index, n (%)					
2017	10 (2.5)	7 (4.0)	1 (1.4)	1 (2.7)	1 (5.0)
2018	62 (15.5)	26 (15.0)	11 (15.1)	6 (16.2)	3 (15.0)
2019	68 (17.0)	31 (17.9)	12 (16.4)	6 (16.2)	6 (30.0)
2020	79 (19.8)	29 (16.8)	13 (17.8)	7 (18.9)	2 (10.0)
2021	75 (18.8)	33 (19.1)	11 (15.1)	3 (8.1)	2 (10.0)
2022	56 (14.0)	19 (11.0)	15 (20.5)	8 (21.6)	3 (15.0)
2023	49 (12.3)	28 (16.2)	10 (13.7)	6 (16.2)	3 (15.0)
Follow-up time from diagnosis in months, median (IQR)	28.0 [15.2, 62.2]	27.6 [18.4, 67.8]	29.9 [18.1, 54.2]	31.7 [19.7, 65.1]	38.0 [22.2, 80.6]
Time from diagnosis to index in months, median (IQR)	16.0 [9.3, 36.0]	20.4 [11.5, 49.3]	22.3 [13.8, 43.3]	27.4 [15.4, 42.2]	31.6 [20.3, 60.6]
Follow-up time from index in months (Reverse KM), median (95% CI)	26.8 (23.7, 33.6)	20.1 (15.9, 50.4)	37.8 (10, NR)	38.5 (26, NR)	28.7 (21.6, NR)
ECOG Status at Index, n (%)					
0–2	217 (54.4)	104 (60.1)	46 (63.0)	23 (62.2)	11 (55.0)
≥3	0 (0.0)	0 (0.0)	0 (0.0)	0 (0.0)	0 (0.0)
Unknown	182 (45.6)	69 (39.9)	27 (37.0)	14 (37.8)	9 (45.0)
Stage at Diagnosis by Any Staging System, n (%)					
1	19 (4.8)	4 (2.3)	1 (1.4)	2 (5.4)	0 (0.0)
2	65 (16.3)	34 (19.7)	13 (17.8)	3 (8.1)	3 (15.0)
3	100 (25.1)	46 (26.6)	23 (31.5)	15 (40.5)	8 (40.0)
4	165 (41.4)	67 (38.7)	28 (38.4)	16 (43.2)	7 (35.0)
Unknown	50 (12.5)	22 (12.7)	8 (11.0)	1 (2.7)	2 (10.0)

**Table 3:** 1L–6L treatment patterns among 3L-qualifying and 4L-qualifying population.

3L-Qualifying (N = 399)	
1L (N = 399)	**n (%)**
CHOP +/− Rituximab +/− Methotrexate	282 (70.7)
Other CHOP-related w/o Polatuzumab	93 (23.3)
Other *	14 (3.5)
Bendamustine-Containing Regimens w/o Lenalidomide	4 (1.0)
Polatuzumab-Containing Regimens	3 (0.8)
ICE or R-ICE	2 (0.5)
Lenalidomide-Containing Regimens	1 (0.3)
2L (N = 399)	**n (%)**
Other *	157 (39.4)
ICE or R-ICE +/− Transplant	71 (17.8)
Bendamustine-Containing Regimens	42 (10.5)
Other Transplant	36 (9.0)
GemOx +/− Rituximab +/− Polatuzumab	29 (7.3)
Investigational Regimens	16 (4.0)
Pola-BR +/− Lenalidomide	13 (3.3)
Tafasitamab-Containing Regimens	12 (3.0)
Lenalidomide-Containing Regimens	11 (2.8)
R2	6 (1.5)
CAR-T	4 (1.0)
Loncastuximab-Containing Regimens	1 (0.3)
Selinexor-Containing Regimens	1 (0.3)
3L (N = 399)	**n (%)**
Other *	168 (42.1)
Pola-BR +/− Lenalidomide	49 (12.3)
GemOx +/− Rituximab +/− Polatuzumab	42 (10.5)
ICE or R-ICE +/− Transplant	33 (8.3)
Lenalidomide-Containing Regimens	33 (8.3)
Bendamustine-Containing Regimens	31 (7.8)
Tafasitamab-Containing Regimens	24 (6.0)
R2	10 (2.5)
Anti-PD-L1- or Anti-PD1-Containing Regimens	5 (1.3)
Loncastuximab-Containing Regimens	4 (1.0)
4L (N = 209)	**n (%)**
CAR-T	59 (28.2)
Other *	51 (24.4)
Investigational Regimens	24 (11.5)
Pola-BR +/− Lenalidomide	18 (8.6)
Tafasitamab-Containing Regimens	14 (6.7)
Lenalidomide-Containing Regimens	12 (5.7)
Bendamustine-Containing Regimens	7 (3.4)
R2	6 (2.9)
ICE or R-ICE +/− Transplant	5 (2.4)
GemOx +/− Rituximab +/− Polatuzumab	3 (1.4)
Loncastuximab-Containing Regimens	3 (1.4)
Selinexor-Containing Regimens	3 (1.4)
BEAM or R-BEAM +/− Transplant	2 (1.0)
Other Transplant	2 (1.0)
5L (N = 82)	**n (%)**
CAR-T	19 (23.2)
Other *	16 (19.5)
Loncastuximab-Containing Regimens	9 (11.0)
Investigational Regimens	6 (7.3)
Tafasitamab-Containing Regimens	6 (7.3)
GemOx +/− Rituximab +/− Polatuzumab	5 (6.1)
Lenalidomide-Containing Regimens	5 (6.1)
Anti-PD-L1- or Anti-PD1-Containing Regimens	4 (4.9)
R2	4 (4.9)
Bendamustine-Containing Regimens	3 (3.7)
ICE or R-ICE +/− Transplant	2 (2.4)
Pola-BR +/− Lenalidomide	2 (2.4)
Other Transplant	1 (1.2)
6L (N = 39)	**n (%)**
Other *	13 (33.3)
Tafasitamab-Containing Regimens	7 (18.0)
CAR-T	4 (10.3)
Investigational Regimens	3 (7.7)
Pola-BR +/− Lenalidomide	3 (7.7)
Anti-PD-L1- or Anti-PD1-Containing Regimens	2 (5.1)
Bendamustine-Containing Regimens	2 (5.1)
Lenalidomide-Containing Regimens	2 (5.1)
Other Transplant	2 (5.1)
Selinexor-Containing Regimens	1 (2.6)
**4L-Qualifying** **(N = 173)**	
1L (N = 173)	**n (%)**
CHOP +/− Rituximab +/− Methotrexate	126 (72.8)
Other CHOP-related w/o Polatuzumab	37 (21.4)
Other *	7 (4.1)
Bendamustine-Containing Regimens w/o Lenalidomide	1 (0.6)
ICE or R-ICE	1 (0.6)
Polatuzumab-Containing Regimens	1 (0.6)
2L (N = 173)	**n (%)**
Other *	71 (41.0)
ICE or R-ICE +/− Transplant	31 (17.9)
Bendamustine-Containing Regimens	20 (11.6)
Other Transplant	13 (7.5)
Investigational Regimens	12 (6.9)
Lenalidomide-Containing Regimens	9 (5.2)
GemOx +/− Rituximab +/− Polatuzumab	7 (4.1)
Pola-BR +/− Lenalidomide	4 (2.3)
Tafasitamab-Containing Regimens	2 (1.2)
Anti-PD-L1- or Anti-PD1-Containing Regimens	1 (0.6)
CAR-T	1 (0.6)
R2	1 (0.6)
Selinexor-Containing Regimens	1 (0.6)
3L (N = 173)	**n (%)**
Other *	47 (27.2)
CAR-T	22 (12.7)
ICE or R-ICE +/− Transplant	20 (11.6)
Investigational Regimens	18 (10.4)
Lenalidomide-Containing Regimens	15 (8.7)
Pola-BR +/− Lenalidomide	14 (8.1)
GemOx +/− Rituximab +/− Polatuzumab	12 (6.9)
Other Transplant	8 (4.6)
Bendamustine-Containing Regimens	6 (3.5)
R2	6 (3.5)
Tafasitamab-Containing Regimens	4 (2.3)
Anti-PD-L1- or Anti-PD1-Containing Regimens	1 (0.6)
4L (N = 173)	**n (%)**
Other *	63 (36.4)
Pola-BR +/− Lenalidomide	26 (15.0)
Bendamustine-Containing Regimens	18 (10.4)
Lenalidomide-Containing Regimens	18 (10.4)
Tafasitamab-Containing Regimens	17 (9.8)
ICE or R-ICE +/− Transplant	8 (4.6)
R2	8 (4.6)
GemOx +/− Rituximab +/− Polatuzumab	5 (2.9)
Loncastuximab-Containing Regimens	5 (2.9)
Selinexor-Containing Regimens	3 (1.7)
Anti-PD-L1- or Anti-PD1-Containing Regimens	2 (1.2)
5L (N = 80)	**n (%)**
CAR-T	24 (30.0)
Other *	13 (16.3)
Tafasitamab-Containing Regimens	7 (8.8)
GemOx +/− Rituximab +/− Polatuzumab	6 (7.5)
Investigational Regimens	6 (7.5)
Loncastuximab-Containing Regimens	6 (7.5)
Bendamustine-Containing Regimens	5 (6.3)
Lenalidomide-Containing Regimens	5 (6.3)
Pola-BR +/− Lenalidomide	3 (3.8)
Anti-PD-L1- or Anti-PD1-Containing Regimens	2 (2.5)
ICE or R-ICE +/− Transplant	2 (2.5)
R2	1 (1.3)
6L (N = 31)	**n (%)**
Other *	8 (25.8)
Tafasitamab-Containing Regimens	6 (19.4)
Investigational Regimens	4 (12.9)
CAR-T	3 (9.7)
Anti-PD-L1- or Anti-PD1-Containing Regimens	2 (6.5)
Bendamustine-Containing Regimens	2 (6.5)
Lenalidomide-Containing Regimens	2 (6.5)
Other Transplant	2 (6.5)
GemOx +/− Rituximab +/− Polatuzumab	1 (3.2)
Pola-BR +/− Lenalidomide	1 (3.2)

* Other category includes all less-frequently administered regimens in the LOT, such as CD20-directed bispecific antibodies.

**Table 4:** rwTTNTD and rwOS overall and among subgroups.

	Time to Next Treatment or Death		Overall Survival			
	rwTTNTD Events	Median rwTTNTD, Months (95% CI)	rwOS Events	Median rwOS, Months (95% CI)	30-Month rwOS Events	30-Month rwOS Rate (95% CI)
All-Qualifying LOTs (N = 716)	594	3.3 (3.0, 3.7)	480	8.0 (6.9, 10.6)	469	20% (17%, 24%)
First-Qualifying LOT (N = 476)	389	3.5 (3.2, 4.0)	299	11.4 (9.7, 13.6)	290	25% (21%, 30%)
Last-Qualifying LOT (N = 476)	354	3.6 (3.3, 4.3)	299	6.3 (5.3, 8.0)	292	25% (21%, 31%)
Among All-Qualifying|By LOT						
3L (N = 399)	329	3.4 (3.0, 3.9)	253	11.1 (8.3, 13.6)	245	25% (20%, 31%)
4L (N = 173)	136	3.6 (2.8, 4.3)	111	7.4 (5.8, 11.0)	109	18% (12%, 27%)
5L (N = 73)	62	2.9 (2.5, 3.8)	53	5.5 (4.1, 8.2)	52	9% (4%, 23%)
6L (N = 37)	36	2.2 (1.3, 3.4)	32	4.0 (2.7, 7.0)	32	9% (3%, 27%)
7L (N = 20)	18	3.1 (2.0, 10.2)	18	3.5 (2.3, 14.9)	18	7% (1%, 41%)
Among All-Qualifying|Baseline ECOG Score						
ECOG 0–2 (N = 408)	345	3.3 (2.8, 3.7)	300	6.9 (5.8, 9.1)	295	17% (14%, 22%)
ECOG Missing (N = 308)	249	3.4 (3.0, 3.8)	180	10.6 (7.9, 13.8)	174	24% (18%, 32%)

**Table 5:** Characteristics among all-qualifying index-LOTs by ECOG 0–2 and M/U.

	ECOG 0–2 (N = 408)	ECOG M/U (N = 308)
Age at Initial Dx, median [IQR]	64.0 [55.0, 72.0]	64.0 [54.0, 72.0]
Age at Index, median [IQR]	66.5 [57.0, 75.0]	66.5 [56.0, 76.0]
Sex, n (%)		
Female	163 (40.0)	113 (36.7)
Male	245 (60.0)	195 (63.3)
Race		
Asian	13 (3.2)	13 (4.2)
Black or African American	23 (5.6)	14 (4.5)
White	290 (71.1)	208 (67.5)
Other	73 (17.9)	62 (20.1)
Unknown	9 (2.2)	11 (3.6)
Ethnicity, n (%)		
Hispanic	108 (26.5)	99 (32.1)
Non-Hispanic	285 (69.9)	201 (65.3)
Unknown	15 (3.7)	8 (2.6)
Practice Setting, n (%)		
Academic	147 (36.0)	114 (37.0)
Community	261 (64.0)	194 (63.0)
Year of diagnosis, n (%)		
1994–2003	6 (1.5)	7 (2.3)
2004–2008	14 (3.4)	8 (2.6)
2009–2013	41 (10.0)	23 (7.5)
2014–2018	198 (48.5)	110 (35.7)
2019	64 (15.7)	47 (15.3)
2020	31 (7.6)	35 (11.4)
2021	28 (6.9)	43 (14.0)
2022	26 (6.4)	33 (10.7)
2023	0 (0.0)	2 (0.6)
Year of Index, n (%)		
2017	11 (2.7)	9 (2.9)
2018	79 (19.4)	31 (10.1)
2019	81 (19.9)	45 (14.6)
2020	94 (23.0)	41 (13.3)
2021	60 (14.7)	65 (21.1)
2022	41 (10.0)	62 (20.1)
2023	42 (10.3)	55 (17.9)
Follow-up time from diagnosis in months, median (IQR)	30.7 [17.5, 68.9]	26.8 [16.7, 60.0]
Time from diagnosis to index in months, median (IQR)	20.4 [11.1, 46.3]	17.8 [10.1, 37.4]
Follow-up time from index in months (Reverse KM), median (95% CI)	36.1 (28, 41.5)	17 (15, 24.2)
Stage at Diagnosis by Any Staging System, n (%)		
1	17 (4.2)	9 (2.9)
2	71 (17.4)	50 (16.2)
3	118 (28.9)	76 (24.7)
4	163 (40.0)	128 (41.6)
Unknown	39 (9.6)	45 (14.6)
Calculated IPI at Diagnosis, n (%)		
Low Risk (IPI Score 0–1)	48 (11.8)	29 (9.4)
Low–Intermediate Risk (IPI Score 2)	63 (15.4)	27 (8.8)
High–Intermediate Risk (IPI Score 3)	30 (7.4)	11 (3.6)
High Risk (IPI Score 4–5)	12 (2.9)	4 (1.3)
Unknown	255 (62.5)	237 (76.9)
ECOG Status at Diagnosis, n (%)		
0–2	209 (51.2)	94 (30.5)
≥3	3 (0.7)	4 (1.3)
Unknown	196 (48.0)	210 (68.2)
BCL2, n (%)		
Negative	36 (8.8)	37 (12.0)
Positive	349 (85.5)	227 (73.7)
Unknown	23 (5.6)	44 (14.3)
BCL6, n (%)		
Negative	56 (13.7)	46 (14.9)
Positive	333 (81.6)	219 (71.1)
Unknown	19 (4.7)	43 (14.0)
C-MYC, n (%)		
Negative	123 (30.1)	81 (26.3)
Positive	227 (55.6)	168 (54.5)
Unknown	58 (14.2)	59 (19.2)

## Data Availability

The data underlying this article were provided by COTA, Inc. (now Verana Health) and cannot be shared due to privacy reasons. Summary-level data are provided throughout the manuscript and in the accompanying tables and figures.

## Supplementary Materials

The following supporting information can be downloaded at: https://www.mdpi.com/article/10.3390/jcm15197596/s1, Table S1. Mapping of ECHELON-3 trial criteria to COTA’s real-world database.

## Abbreviations

The following abbreviations are used in this manuscript:

1L	First-line
2L	Second-line
3L+	Third-line or later
BV	Brentuximab vedotin
CAR	Chimeric antigen receptor
CNS	Central nervous system
DLBCL	Diffuse large B-cell lymphoma
ECOG	Eastern Cooperative Oncology Group
EHR	Electronic health record
GemOx	Gemcitabine + oxaliplatin
HSCT	Hematopoietic stem cell transplant
IPI	International Prognostic Index
IQR	Interquartile range
KM	Kaplan–Meier
LOT	Line of therapy
M/U	Missing/unknown
NHL	Non-Hodgkin lymphoma
OS	Overall survival
Pola-BR	Pola-bendamustine + rituximab
Pola-R-CHP	Polatuzumab vedotin, rituximab, cyclophosphamide, doxorubicin, and prednisone
R2	Lenalidomide and rituximab
R/R	Relapsed/refractory
R-CHOP	Rituximab, cyclophosphamide, doxorubicin, vincristine, and prednisone
rw	Real-world
SOC	Standard of care
TTNTD	Time to next treatment or death
