# Supplementary material for: Analysis of Treatment Patterns and Outcomes Among Patients with Third-Line or Later (3L+) Diffuse Large B-Cell Lymphoma (DLBCL) with Multiple Qualifying Index Dates Treated in the United States
Source: J Clin Med. 2026 Sep 30;15(19):7596. doi: 10.3390/jcm15197596 (PMC13665813; doi:10.3390/jcm15197596)
Supplement: Supplementary file 1 [file jcm-15-07596-s001.zip › jcm-4552741-supplementary.pdf]

**Table S1. Mapping of ECHELON-3 trial criteria to COTA's real-world database.**

| ECHELON-3 Trial Inclusion Criteria | Proposed RWE I/E criteria to apply |
|---|---|
| • Participants with relapsed or refractory diffuse and transformed large B-cell lymphoma (R/R DLBCL). DLBCL and cell of origin (GCB versus non-GCB) will be histologically determined by local pathology assessment for the purposes of study eligibility and stratification. | Patients must have histologically or pathologically confirmed DLBCL, received treatment with at least two prior lines of therapy, and have a record of initiating third-line therapy during the index period. |
| • Participants must have R/R disease following 2 or more lines of prior systemic therapy.<br>o For participants with transformed DLBCL, at least the last systemic therapy used must have been for DLBCL. | |
| • Participants must be HSCT or CAR-T ineligible according to the investigator and must meet at least one of the following criteria:<br>1. One or more co-morbidities, including cardiac, pulmonary, renal, or hepatic dysfunction, that, in the opinion of the Investigator, make the participant medically unfit to received HSCT or CAR-T therapy;<br>2. Active disease following induction and salvage chemotherapy;<br>3. Inadequate stem cell mobilization (for HSCT);<br>4. Relapse following prior HSCT or CAR-T;<br>5. Unable to receive CAR-T therapy due to financial, geographic, insurance, or manufacturing issues. | Patients must not receive HSCT or CAR-T cell therapy in the potential index LOT. |
| • Participants must have tumor tissue submitted to the central pathology lab. The tumor tissue submitted should be from the most recent biopsy that contains DLBCL. | Not applicable; known CD30 status was not required but was captured when documented. |
| • An Eastern Cooperative Oncology Group (ECOG) performance status score of 0 to 2. | Patients must not have an ECOG score of 3 or greater within the baseline period of the potential index LOT. |
| • Participants must have fluorodeoxyglucose (FDG)-avid disease by positron emission tomography (PET) and bidimensional measurable disease of at least 1.5 cm by computed tomography (CT), as assessed by the site radiologist within 28 days of Day 1. | Not applicable in real-world data. |
| | |
| **ECHELON-3 Trial Inclusion Criteria** | **Proposed RWE I/E criteria to apply** |
| • History of another malignancy within 2 years before the first dose of study drug or any evidence of residual disease from a previously diagnosed malignancy. | Patients must not have a diagnosis of a solid tumor cancer/malignancy (excluding non-malignant skin cancer, non-Hodgkin lymphoma, or leukemia) documented within two years prior to potential index date. |
| • History of progressive multifocal leukoencephalopathy (PML). | Due to low prevalence in real-world population and |

| ECHELON-3 Trial Inclusion Criteria | Proposed RWE I/E criteria to apply |
|---|---|
| | lack of available data, this criterion was not applied. |
| • Active cerebral/meningeal disease related to the underlying malignancy. Participants with a history of cerebral/meningeal disease related to the underlying malignancy are allowed if prior CNS disease has been effectively treated and without progression for at least 3 months. | Patients must not have received radiation to a CNS site or had a positive CNS biopsy prior to the potential index date. |
| • Any uncontrolled Grade 3 or higher (per NCI CTCAE version 5.0) viral, bacterial, or fungal infection within 2 weeks prior to the first dose of study drug. Routine antimicrobial prophylaxis is permitted. | Not applicable; this trial criterion is related to patient safety and trial validity, which is not a concern for the real-world study. |
| • Chemotherapy, radiotherapy, biologics, and/or other antitumor treatment with immunotherapy that is not completed 3 weeks prior to first dose of study drug, unless underlying disease has progressed on treatment. | Not applicable; this trial criterion is related to patient safety and trial validity, which is not a concern for the real-world study. |
| • Previous treatment with brentuximab vedotin or lenalidomide. Previous treatment with other vedotin-based ADCs is permitted if the last dose is at least 6 months prior to Day 1. | Patients must not receive brentuximab vedotin at or any time prior to the potential index LOT. |
| • Current therapy with immunosuppressive medications (including steroids), other systemic anti-neoplastic, or investigational agents;<br>a) Prednisone (or equivalent) ≤10 mg/day may be used for non-lymphomatous purposes. | Patients must not receive investigational therapy at the potential index LOT. |
| • Documented history of a cerebral vascular event (stroke or transient ischemic attack), unstable angina, myocardial infarction, or cardiac symptoms consistent with New York Heart Association (NYHA) Class III-IV within 6 months prior to the first dose of study drugs. | Not applicable; this trial criterion is related to patient safety and trial validity, which is not a concern for the real-world study. |
| • Congestive heart failure, Class III or IV, by the NYHA criteria. | Not applicable; this trial criterion is related to patient safety and trial validity, which is not a concern for the real-world study. |
| • Grade 2 or higher peripheral sensory or motor neuropathy at baseline. | Not applicable; this trial criterion is related to patient safety and trial validity, which is not a concern for the real-world study. |
